# Biomonitoring of toxic metals in feathers of predatory birds from eastern regions of Hungary

**DOI:** 10.1007/s11356-019-05723-9

**Published:** 2019-07-09

**Authors:** Adrienn Grúz, Oliver Mackle, András Bartha, Rita Szabó, János Déri, Péter Budai, József Lehel

**Affiliations:** 1 Department of Hygiene, Institute of Plant Protection, Georgikon Faculty, University of Pannonia, Deák F. u. 16, Keszthely, H-8360 Hungary; 2 Department of Food Hygiene, University of Veterinary Medicine, István u. 2., Budapest, H-1400 Hungary; 3 Department of Animal Hygiene, Herdhealth and Veterinary Ethology, University of Veterinary Medicine, István u. 2., Budapest, H-1400 Hungary; 4 Bird Hospital Foundation, Petőfi tér 6., Hortobágy, H-4071 Hungary

**Keywords:** Environmental contaminants, Heavy metals, Predatory birds, Feathers, Monitoring, Pollution, Owls, Buzzards, Common kestrels, Eurasian sparrow-hawks

## Abstract

The aim of our study was to investigate the concentrations of toxic metals in the feathers of predatory birds in the Hortobágyi Madárpark (Bird Hospital Foundation). Samples were collected from different predatory birds originated from the eastern and north-eastern region of Hungary. Inductively coupled plasma optical emission spectrometry was used to determine the concentration of toxic metals. The mean values varied between bird species groups, their concentrations were between 0.29 ± 0.24 and 0.40 ± 0.30 mg/kg for arsenic (As), 0.09 ± 0.03 and 0.20 ± 0.18 mg/kg for cadmium (Cd), 1.15 ± 1.40 to 2.30 ± 1.52 mg/kg for lead (Pb) and 0.58 ± 0.31 to 2.19 ± 1.25 mg/kg for mercury (Hg), respectively. The measured values are not over the considered threshold values for these toxic metals and in accordance with similar concentrations of them recorded in similar species within Europe. No significant differences were found in their concentration between genders or age in the species. According to the detected concentrations of these metals, their levels accumulated in the feather of the investigated birds do not indicate the possibility of poisoning.

## Introduction

In recent years, toxic metals have become a great concern for human, animal and environmental welfare due to the pollution arising from increased intensity of industrial, commercial, mining and agriculture production. Levels of heavy metals have been increased in the environment because of these anthropogenic activities (Markowski et al. 2013).

The toxic metals most commonly associated with poisoning of humans and animals are arsenic (As), cadmium (Cd), lead (Pb) and mercury (Hg). Toxic metal poisoning may occur from industrial exposure, water or atmosphere pollution, food sources, medicines, improperly coated food containers or the ingestion of Pb-based paints.

Accumulation of toxic metals in feedstuffs destined for human or livestock animal consumption can be easily checked and monitored whereas the diet of wild animals is almost impossible to regulate. Heavy metals have been shown to accumulate in kidney, liver, blood, feathers, eggs and bones (Burger and Gochfeld 1993; Fasola et al. 1998; Mateo and Guitart 2003; Deng et al. 2007; Jayakumar and Muralidharan 2011; Farahani et al. 2015; Kitowski et al. 2016; Zarrintab and Mirzaei 2018).

Due to the accumulation property of toxic metals in wild birds’ tissues, they can be used as bio-indicators for environmental contamination. Bird’s feathers have been used in previous studies and have been shown to be appropriate bio-indicators of metal pollution. Feathers of predatory birds have proved to be good indicators of the status of environmental heavy metal pollution (Carneiro et al. 2015) because they occupy higher trophic levels within an ecosystem, are long-lived, sensitive to atmospheric environmental changes and reveal compounds which bio-accumulate in prey such as methylmercury (Monteiro and Furness 1995; Burger 2002). Furthermore, they are easy to sample from live, dead or museum specimens and can be easily removed from live birds without causing damage and give a chance to examine also endangered species. Besides, the storage of feathers is easy because they do not need to keep in refrigerator and can be collected more times from the same bird and over large geographical area (Walsh 1990; Burger 1993, 1996; Movalli 2000, Abdullah et al. 2015; Ansara-Ross et al. 2013; Kim and Oh 2014; Rubio et al. 2016).

Collecting feather samples is a non-invasive way to collect tissue samples from birds. It had been found to be representative of toxic metal burden in the body, since the feathers being moulted yearly, it allows these samples to represent the exposure of these birds in the previous year, and this can provide a useful and non-destructive tissue. For example, in the case of Hg, birds excrete substantial level of different metals in the feathers, where the rate is nearly constant for Hg, and relatively high for certain metals (Burger 1993; Burger and Gochfeld 2000).

Moreover, a strong correlation had been determined between levels of Hg in the diet and the feathers of birds, which provides another advantage for collecting data with the analysis of feathers for estimating the contamination of the birds (Monteiro et al. 1998).

The aim of this study was to measure and evaluate the As, Cd, Pb and Hg concentration in Long-eared owl (*Asio otus*), Barn owl (*Tyto alba*), Tawny owl (*Strix aluco*), Little owl (*Athene noctua*), Buzzards (*Buteo buteo*), Common kestrels (*Falco tinnunculus*) and Eurasian sparrow-hawks (*Accipiter nisus*), collected from around Hungary. For this, feather samples were taken from them and their heavy metal contents were detected by inductively coupled plasma optical emission spectrometry.

## Materials and methods

### Feathers

**Fig. 1:**
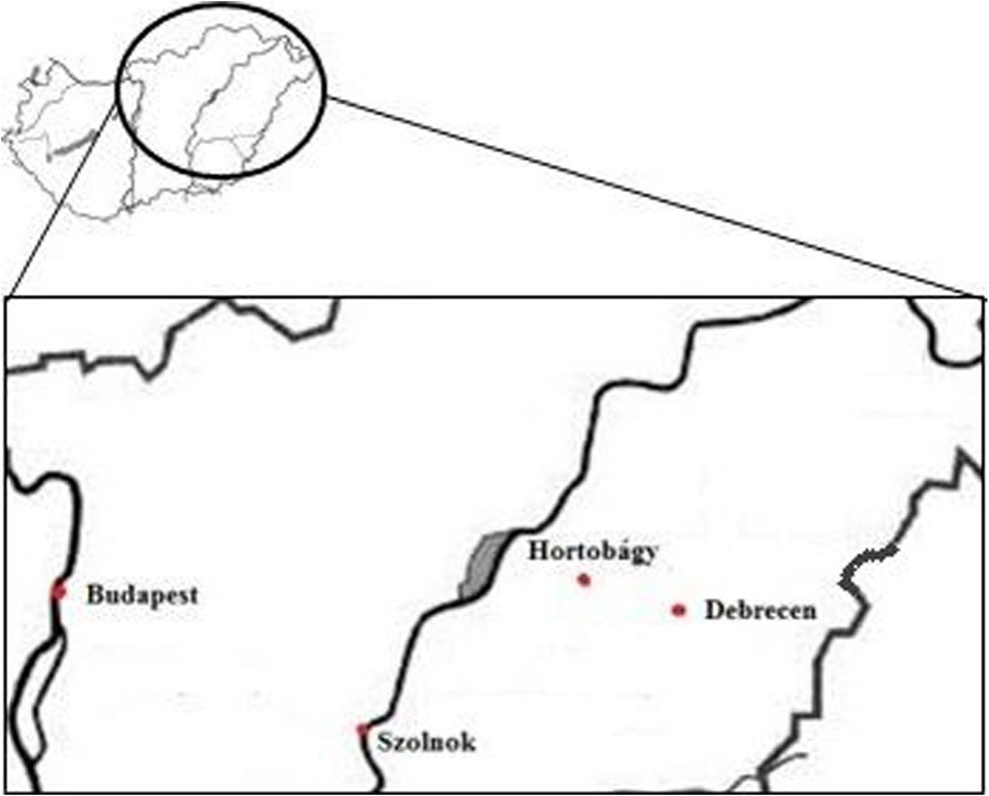
Founding location of bird species (by Adrienn Grúz)

The samples were collected in the Hortobágyi Madárpark (Bird Hospital Foundation), Hortobágy, Hungary, where the birds arrived mostly for medical treatment from the eastern and north-eastern region of Hungary (Fig. 1). The examined region is geographically varied (from mountainous with woody habitats to steppe with sand-hills and rivers). Agricultural areas and possible environmental polluting factories, such as oil refinery, drug company, fertilizer warehouse, chemical and incineration plant, can be found in the area with 100 km distance.

Different data were noted during the collection as follows: time and location of finding, gender, and birds’ age. Determination of the age was carried out by the colour of their plumage and the size of the bird, and for example in the case of the buzzards, the colour of their eyes is different in the first 2 years (in the first year is livid, in the second year is yellow and from the third year is brown).

A mixed sample of primaries, secondaries and coverts were collected by plucking, from predatory birds including 41 Owls—Long-eared owl (28; 9 juveniles and 14 adults, 5 without age), Barn owl (2; 2 juveniles), Tawny owl (2, 2 adults), Little owls (8 juveniles and 1 adult), 40 Buzzard (18 juveniles and 18 adults, 4 without age), 18 Common kestrels (7 juveniles, 10 adults, 1 without age) and 24 Eurasian sparrow-hawks (9 juveniles, 13 adults and 2 without age).

All the collected samples were stored at a dry, well-ventilated place till the analysis.

### Analytical method

Before the analytical procedure to remove adherent exogenous contamination, the feathers were washed with de-ionized water and acetone. Half gram from each sample was weighed individually into a CEM MARS6 MARSXPreSS teflon vessel for digestion. Then, samples were decomposed by 5 mL nitric acid (69 m/m%) and 5 mL hydrogen peroxide (30% m/m%) by microwave digestion system (Ramp: 35 min; Temperature: 200 °C; hold: 50 min; E: 1700 W).

The sample was filled up with ultrapure water to 25 mL and analysed by inductively coupled plasma optical emission spectrometry (ICP-OES) after a double dilution by de-ionized water using 1-mg/L Y solution as internal standard and 0.25 mg/L Au for the stabilization of Hg content. Blank and the quality control (QC) samples were prepared by the same method. Internal quality control of the measurements was carried out via measuring QC samples of known heavy metal concentration at least 10 times (NIST 1577C-standard bovine liver). After discarding the extremes, the standard deviation of data (SD) was established, which must has remained within the ± 15% of the nominal concentration value in order to accept the QC measurement. Every samples, calibration and blank solutions were analysed by three replicates.

Determination of toxic metals was carried out by a Perkin Elmer Optima 8300 DV ICP-OES instrument. Calibration range was between 0 and 200 mg/kg. The detection limits were the followings: As, Hg: 0.20 mg/kg, Cd: 0.02 mg/kg, Pb: 0.10 mg/kg.

The validation of the method was performed as described by Grúz et al. (2018).

### Statistical analysis

Statistical analysis was used to evaluate the results and was looked to answer some key questions regarding the toxic metal burden accumulating in predatory birds based on comparisons of (1) difference between male and female birds, (2) difference between adult and juvenile birds and (3) correlation between the four heavy metals that have been tested.

The statistical analysis of the data was performed using the Software: R version 3.4.2. Wilcoxon rank-sum test was used to compare distributions. Comparisons between age and gender were performed independently. Individuals with unknown age or gender were excluded from the analysis. All data were included in the calculation of correlation between the toxic metals, age and gender. Because data were not normally distributed and in few cases the concentration of the metals was under the detection limit, Spearman rank correlation was calculated and tested. *P* value < 0.05 was significant. The average and standard deviation for both sexes and age groups have been calculated separately using Microsoft Excel.

## Results

The average concentrations of the tested heavy metals for Owls, Buzzards, Common kestrels and Eurasian sparrow-hawks are presented in Table 1.

**Table 1:** Toxic metal concentration of the predatory birds (mean ± SD, mg/kg)

Species	*n*	Arsenic	Cadmium	Mercury	Lead
Owl species					
Males	17	0.32 ± 0.38	0.08 ± 0.04	0.68 ± 0.31	2.25 ± 0.80
Females	19	0.40 ± 0.17	0.08 ± 0.05	0.52 ± 0.35	2.12 ± 1.74
Adult	17	0.36 ± 0.25	0.09 ± 0.05	0.58 ± 0.34	2.10 ± 1.36
Juvenile	19	0.46 ± 0.39	0.12 ± 0.21	0.60 ± 0.27	2.39 ± 1.77
All birds	41	0.40 ± 0.30	0.10 ± 0.14	0.58 ± 0.31	2.30 ± 1.52
Buzzard					
Males	18	0.33 ± 0.17	0.08 ± 0.03	0.63 ± 0.27	0.83 ± 0.53
Females	17	0.32 ± 0.14	0.09 ± 0.03	0.67 ± 0.43	1.67 ± 2.01
Adult	18	0.40 ± 0.28	0.09 ± 0.03	0.69 ± 0.34	1.16 ± 0.59
Juvenile	18	0.28 ± 0.13	0.78 ± 0.04	0.63 ± 0.36	1.20 ± 1.99
All birds	40	0.33 ± 0.17	0.09 ± 0.03	0.64 ± 0.35	1.15 ± 1.40
Common kestrel					
Males	8	0.20 ± 0.00	0.13 ± 0.10	0.64 ± 0.37	2.02 ± 1.30
Females	9	0.36 ± 0.32	0.25 ± 0.20	0.56 ± 0.37	2.50 ± 1.81
Adult	10	0.30 ± 0.30	0.21 ± 0.19	0.46 ± 0.29	1.85 ± 1.24
Juvenile	7	0.30 ± 0.16	0.20 ± 0.18	0.75 ± 0.36	2.48 ± 2.02
All birds	18	0.29 ± 0.24	0.20 ± 0.18	0.59 ± 0.36	2.10 ± 1.57
Eurasian sparrow-hawk					
Males	11	0.25 ± 0.12	0.12 ± 0.11	2.09 ± 1.39	2.19 ± 1.10
Females	11	0.40 ± 0.21	0.10 ± 0.05	2.44 ± 1.10	1.69 ± 0.52
Adult	13	0.37 ± 0.21	0.12 ± 0.10	2.29 ± 1.21	2.06 ± 1.06
Juvenile	9	0.27 ± 0.12	0.09 ± 0.06	2.30 ± 1.31	1.70 ± 0.64
All birds	24	0.32 ± 0.18	0.11 ± 0.09	2.19 ± 1.26	1.84 ± 0.92
*n* number of sample					

Using the Wilcoxon rank sum test, there were no significant differences between age or gender for any of the tested metals in all investigated bird species.

The results regarding the correlation of toxic metal concentration in the predatory bird’s feathers studied had some positive correlations (Table 2).

**Table 2:** Results of Spearman’s rank correlation (rho, *p* value)

Species	Metals	ρ(rho)	*p* value
Owl species			
	As-Cd	0.43	0.01
	As-Hg	0.02	0.88
	As-Pb	− 0.2268	0.16
	Cd-Hg	0.18	0.25
	Cd-Pb	0.38	0.02
	Hg-Pb	0.13	0.42
Buzzard			
	As-Cd	0.02	0.90
	As-Hg	0.21	0.19
	As-Pb	0.20	0.21
	Cd-Hg	0.14	0.38
	Cd-Pb	0.51	0.00
	Hg-Pb	0.06	0.73
Common kestrel			
	As-Cd	− 0.11	0.67
	As-Hg	− 0.32	0.20
	As-Pb	0.17	0.51
	Cd-Hg	− 0.14	0.57
	Cd-Pb	− 0.25	0.31
	Hg-Pb	0.22	0.39
Eurasian sparrow-hawk			
	As-Cd	0.17	0.44
	As-Hg	− 0.17	0.44
	As-Pb	0.02	0.94
	Cd-Hg	0.02	0.91
	Cd-Pb	0.49	0.01
	Hg-Pb	0.39	0.06

**Fig. 2:**
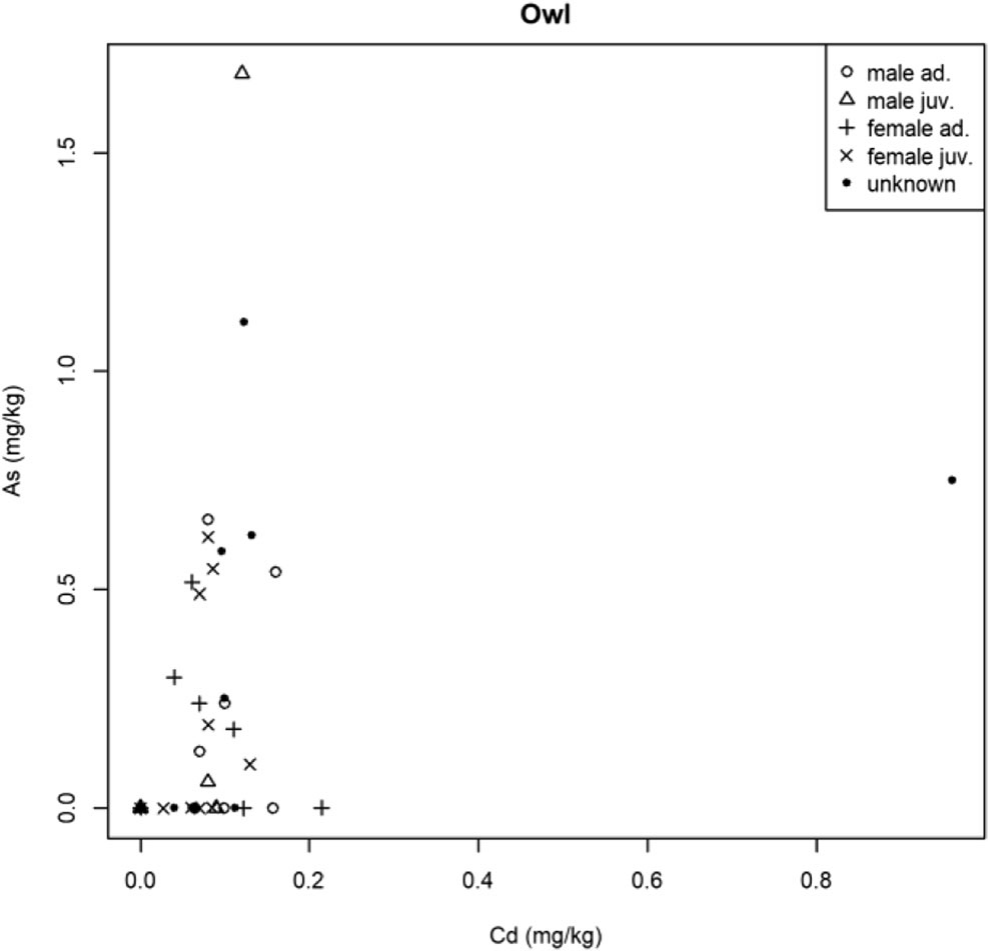
Correlation of As and Cd in Owls

**Fig. 3:**
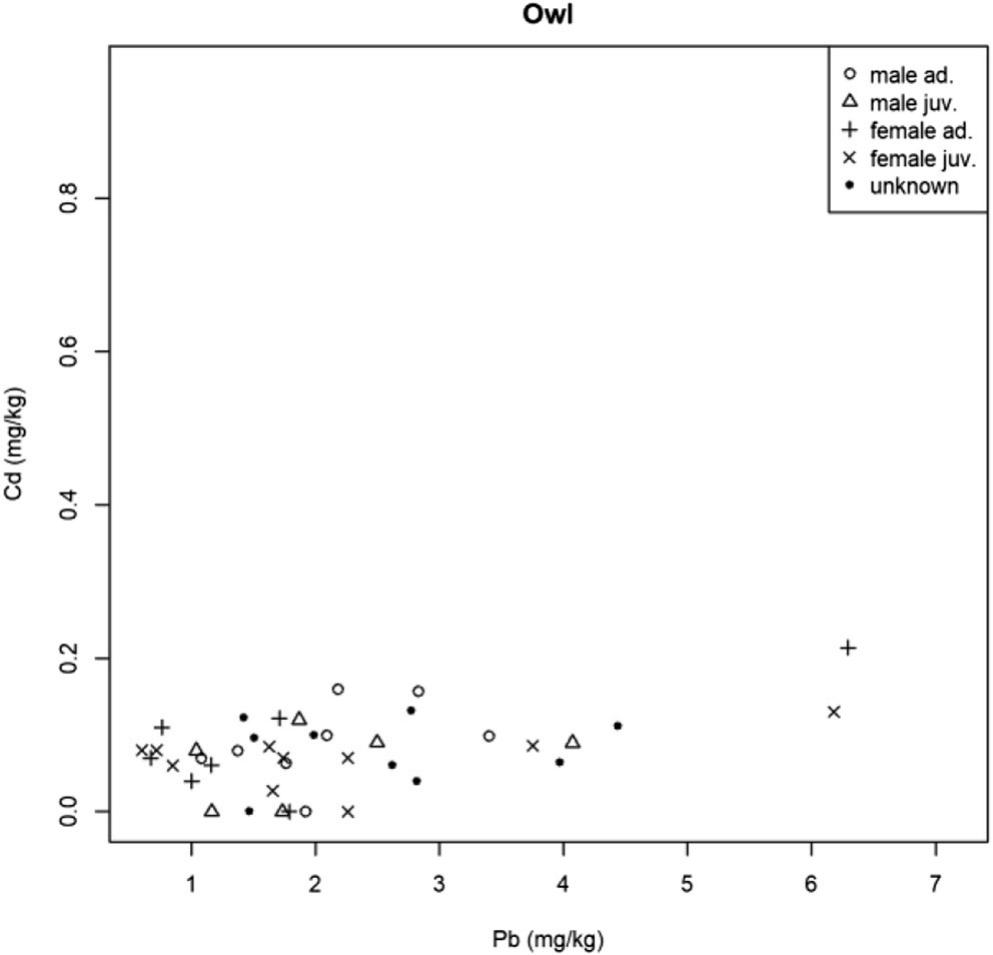
Correlation of Cd and Pb in Owls

In the Owls studied, Cd was significantly correlated with As (Fig. 2) and Pb (Fig. 3), the Spearman rank-correlation coefficient was 0.43 and 0.38, respectively; the connection was medium in both cases.

**Fig. 4:**
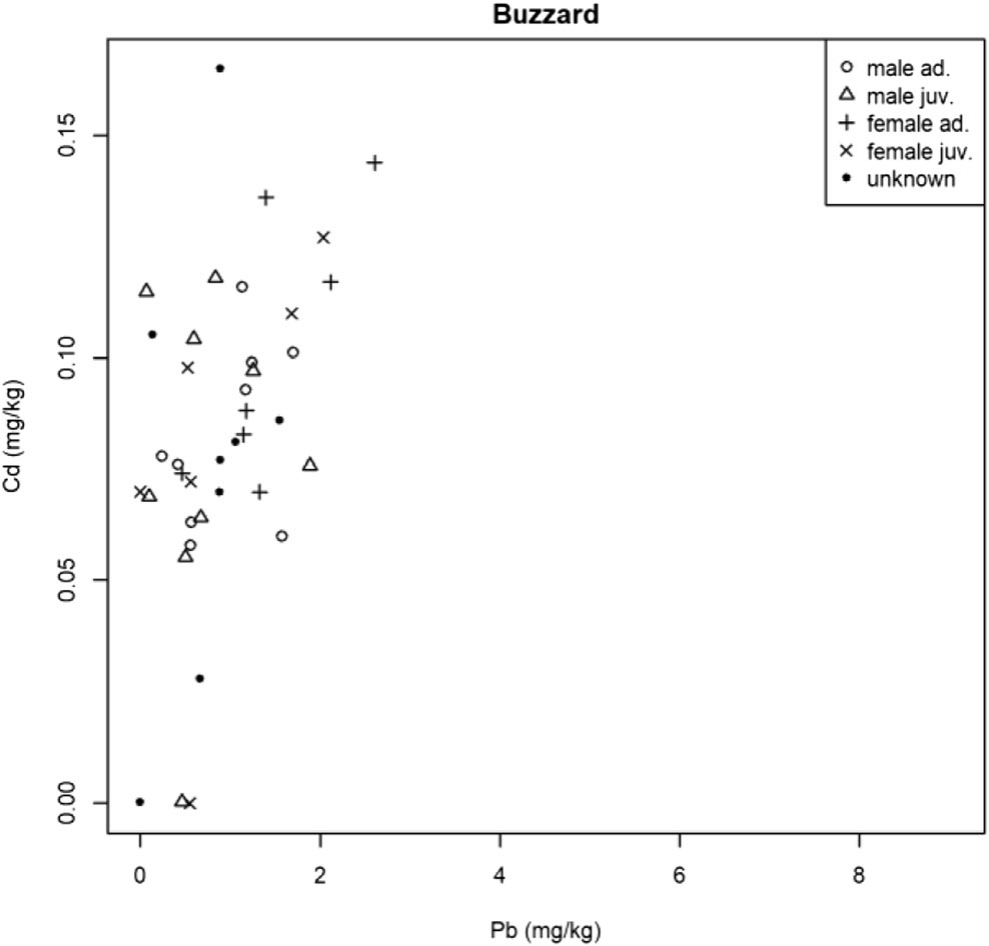
Correlation of Cd and Pb in Buzzards

In the Buzzards studied, a significant correlation was found between Cd and Pb (Fig. 4). The Spearman rank coefficient was 0.51 and the connection was medium.

**Fig. 5:**
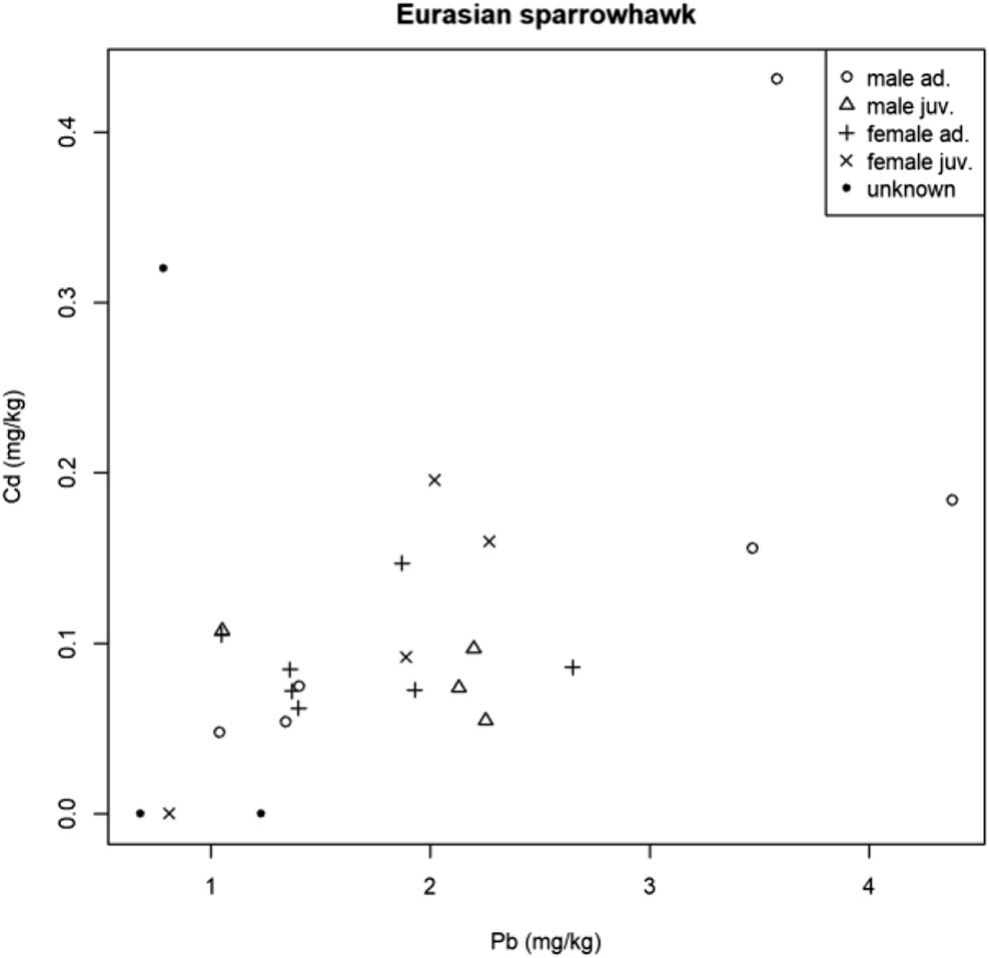
Correlation of Cd and Pb in Sparrow-hawks

There was no significant correlation between any of the heavy metals examined in the case of common kestrels. In the Eurasian sparrow-hawks’ samples analysed, Cd was significantly correlated with Pb (Fig. 5) having a Spearman rank-correlation coefficient of 0.49, the connection was medium. In the case of the Eurasian sparrow-hawks’ samples between Hg and Pb, a medium correlation can be seen, but by the *p* value, this is not statistically significant, what can be caused by the small sample size. So, the understanding is that we do not have sufficient evidence to suggest that there is a correlation between these metals.

## Discussion

The possibility of biomagnification of toxic metals in the food chain is getting well-known nowadays, because before it was only known for persistent pesticides (Laskowski 1991). Birds, especially raptor species are on the top of the food chain and their territorial and non-migratory behaviour also contribute to the accumulation of toxic metals with a higher concentration, in their body (tissues, bones, and feathers) and eggs. Thus, it makes them a good bio-indicator for detecting the environmental burden of heavy metals (Wayland and Bollinger 1999; Zaccaroni et al. 2003).

The individual samples that had increased concentrations of toxic metals may have come from the diet of the bird, not because of external contamination of the feather, since the first step of the analytical procedure is to wash the external contamination.

As can be found in the environment as organic and inorganic forms and induces anaemia, liver damage, cancer and weight loss (Webb 1966; Nemery 1990; Abernathy et al. 1999; Duker et al. 2005).

As can accumulate in the body of animals through the contaminated drinking water or food (Mandal 2017).

The highest concentration of As in the four species groups studied was 0.40 ± 0.30 mg/kg in Owls, which is significantly lower than the concentration of 5 mg/kg found in the USA in an area of possible As pollution (Wiemeyer et al. 1980). There are few relatable studies that use feathers to analyse the quantity of As which makes it difficult in establishing whether the concentration of As recorded in this study is significant or not (Burger et al. 2015). Nighat et al. (2013) measured 1.06–6.44 mg/kg As concentration in four species from Strigidae family. In another study, 0.12 and 0.44 mg/kg As was measured in calamuses and vanes of cinereous vulture (*Aegypius monachus L.*), which is also considered to be very low level like in our study (Yamac et al. 2019).

Cd is categorized as one of the most dangerous elements with long persistence in the environment and high toxicity (Battaglia et al. 2003) and affects the endocrine system, kidneys, reproduction, moulting, haemoglobin formation and growth (Nordberg 1971; Cheney et al. 1981; Eisler 1988; Stoica et al. 2000; Hui 2002). Birds can uptake Cd through terrestrial food, e.g. when the Cd is present in the soil (and air), it can deposit in plants, eaten by different animals and when the meat of these animals are eaten by the raptor species (Van Assche 1998). It can be a risk for secondary poisoning, depending on the concentration of the Cd. The threshold value for Cd in birds is accepted as 3 mg/kg in liver (Scheuhammer 1987; Nighat et al. 2013) that suggests an increased environmental level, but our concentrations measured are much lower than this value at 0.10 ± 0.14 mg/kg in Owls, 0.09 ± 0.03 mg/kg in Buzzards, 0.20 ± 0.18 mg/kg in the Common kestrels and 0.11 ± 0.09 mg/kg in Eurasian sparrow-hawks. Yamac et al. (2019) measured 0.25 ± 1.59 mg/kg Cd in the calamus, and a higher level in the vane 0.33 ± 0.16 mg/kg) of the feather of cinereous vulture. Besides these data, Cd concentrations up to 1.6 mg/kg in raptor bird feathers were measured in an area considered as unpolluted by Lodenius and Solonen (2013). According to data from other studies analysing heavy metals in birds of prey (Naccari et al. 2009), adverse effects have been found at lower levels ranging from 0.1 to 2 mg/kg (Burger 1993), such as reduced growth rates (Spahn and Sherry 1999). So, it can be possible that the sampled birds in this study are experiencing mild adverse effects because of the Cd burden in the environment, even if the area is not heavily polluted.

Anthropogenic sources of Hg are from the burning of fossil fuels, production of steel and phosphate and mining for gold. Animals can take it up by plants grown on contaminated soils or feeding from poisoned meat. Since Hg can accumulate faster in the body tissues than excrete, and also it has a property of biomagnification, it can cause a higher concentration in the body of animals on higher trophic levels (NRC 2005).

The threshold for toxicity of Hg in bird species is highly variable amongst difference species. Sublethal levels from 2.40 mg/kg have been shown to cause impairments in the reproductive processes (Scheuhammer et al. 2007; Jackson et al. 2011). Mashroofeh et al. (2015) compared the Hg concentration in bird feathers from five different trophic levels, and their results show that the carnivore bird feathers (Hen harrier (*Circus cyaneus*): 0.80 ± 0.15 mg/kg; Marsh harrier (*C. aeruginosus*): 1.10 ± 0.20 mg/kg) accumulate more Hg, than piscivore, benthivore, omnivore or herbivore. Roque et al. (2016) reported 1.4 mg/kg Hg in the primary feathers of Barn Owls. It can be explained by the position of the birds in the food chain and their diet. Similarly to our results, Solonen and Lodenius (1990) measured higher Hg concentration in the feather of bird-eater sparrow-hawk, than in mostly rodent-eater species, e.g. owls.

Our values recorded are much lower at 0.58 ± 0.31 mg/kg in Owls, 0.64 ± 0.35 mg/kg in Buzzards and 0.59 ± 0.36 mg/kg in the Common kestrels. But the highest concentration of Hg was recorded in the sample of Eurasian sparrow-hawks, the average was 2.19 ± 1.25 mg/kg. This result is similar to our previous findings (Grúz et al. 2018), where the Hg concentration in Sparrow-hawk feathers was 2.72 mg/kg and it was much higher, compared to the other species, what we examined. In our recent study, the highest individual concentration was 5.43 mg/kg which is a level that could be considered to impact on the reproductive performance of that individual. Hg accumulates in the higher trophic levels of the food chain and the Eurasian sparrow-hawk’ diet may explain the higher concentrations recorded compared to the three other species groups studied.

Adverse effects of Pb concentration can be observed above 4 mg/kg (Burger and Gochfeld 2000) and the considered threshold level for Pb is 2 mg/kg in liver (Pain et al. 1995). Analysing the different parts of the feather of cinereous vulture, Yamac et al. (2019) measured 0.65 mg/kg Pb in the calamus, and 5.47 mg/kg Pb in the vane. The average value that was recorded in our study was between 1.15 and 2.3 mg/kg which is low enough that adverse effects should not be observed in these birds. Although there were 5 Owls, 1 Buzzard, 3 Common kestrels and 1 Eurasian sparrow-hawk sample over the threshold value which could be arising from their food as a cause of secondary poisoning or other common sources of Pb. Major sources of Pb for animals are foodstuffs from contaminated soil. Since plants do not take up Pb easily, mostly they are contaminated exogenously. Furthermore, pollution can come from anthropogenic sources, e.g. from smelters and chemical plants; however, the biomagnification does not typically occur in the higher trophic levels, and a high level of Pb in organisms is associated with proximity to contaminated sites.

Another cause can be the secondary poisoning that occurs when raptors eat birds or other animals what are weak because of poisoning or feeding from Pb poisoned carcasses (Lee 2003; Legagneux et al. 2014).

There was no significant difference in the concentration of the toxic metals due to gender in the four species groups that have been analysed in our study. Several other studies that have found gender difference have also concluded that there is little difference in the concentration of heavy metals between the males and the females (Esselink et al. 1995; Movalli 2000; Zaccaroni et al. 2003) and any difference found was attributed to physiological and ecological differences. Lower levels of metals in female buzzards have been found (Naccari et al. 2009) that they refer to the excretion of heavy metals in the eggs of those birds, but we found no such difference in this study.

Burger et al. (1994) examined breast feathers of Common terns (*Sterna hirundo*) and found no age-related difference in the concentration of Hg, Pb and Cd which supports the results that have been showed in the study relating to the comparison of age groups.

Similarly, no correlation was found between adults and juveniles of investigated bird species in our study. It would be expected that the concentration of heavy metals would increase with age as juvenile birds would have had less exposure time to pollution of the environment but the older birds have also had seasons to excrete heavy metals during their moulting of feathers (Burger 1993).

Regarding the correlation of toxic metals at our investigated area, significant correlation was recorded in the Owls, Buzzards and Eurasian sparrow-hawks between the concentration of Cd and Pb, and in the Owls between As and Cd. Similar results were reported in the case of significant correlation between Cd and Pb concentration in feathers by Zarrintab et al. (2016) and in the calamus by Yamac et al. (2019) and between As and Cd concentration in the vane (Yamac et al. 2019). It can be explained with the region from which the birds inhabited where both agricultural and industrial area can be found. For example, Pb is especially associated with Pb ammunition using during hunting and as it was only banned in 2005, it is very possible that still there is Pb ammunition in the environment of these birds. It would be expected that the levels of Pb and Cd are in decline as their use in anthropogenic source such as Cd batteries and leaded fuels have also declined (ATSDR 1999). Sparse literature is available on the study of metals in birds within the sampled region, from which trends over time in the concentration of the toxic metals could be observed.

## Conclusions

Even though there was no correlation between the ages and the sexes, it can be said that bird feather testing is a useful method for monitoring the presence of toxic metals which can be found in the environment and can be accumulated in birds. As it can be seen by our data and based on other studies, the concentration of these toxic metals measured in the feathers stays below the level that can cause adverse or toxicological effects in birds.

## References

[CR1] Abdullah M, Fasola M, Muhammad A, Malik SA, Boston N, Bokhari H, Kamran MA, Shafqat MN, Alamdar A, Khan M, Ali N, Eqani SAMAS (2015). Avian feathers as a non-destructive bio-monitoring tool of trace metals signatures: a case study from severely contaminated areas. Chemosphere.

[CR2] Abernathy CO, Y-Liu D, Longfellow HV, Aposhia B, Beck B, Fowler R, Goyer R, Menzer T, Rossman C, Thompson M, Waalkes (1999). Arsenic: health effects, mechanisms of actions, and research issues. Environ Health Perspect.

[CR3] Ansara-Ross TM, Ross MJ, Wepener V (2013). The use of feathers in monitoring bioaccumulation of metals and metalloids in the south African endangered African grass-owl (Tyto capensis). Ecotoxicology.

[CR4] ATSDR (Agency for Toxic Substances and Disease Registry) (1999). Toxicological profile for cadmium.

[CR5] Battaglia A, Ghidini S, Campanini G, Spaggiari R (2003). Heavy metal contamination in little owl (*Athene noctua*) and common buzzard (*Buteo buteo*) from northern Italy. Ecotoxicol Environ Saf.

[CR6] Burger J (1993). Metals in avian feathers: bioindicators of environmental pollution. Rev Environ Contam Toxicol.

[CR7] Burger J (1996). Heavy metal and selenium levels in feathers of franklin’s gulls in interior North America. Auk.

[CR8] Burger J (2002) Food chain differences affect heavy metals in bird eggs in Barnegat Bay, New Jersey. Environmental Research 90:33–39 10.1006/enrs.2002.4381 12359188

[CR9] Burger J, Gochfeld M (1993). Heavy metal and selenium levels in feathers of young egrets and herons from Hong Kong and Szechuan, China. Arch Environ Contam Toxicol.

[CR10] Burger J, Gochfeld M (2000). Metals in albatross feathers from midway atoll: influence of species, age, and nest location. Environ Res.

[CR11] Burger J, Nisbet ICT, Gochfeld M (1994). Heavy metal and selenium levels in feathers of known-aged common terns (*Sterna hirundo*). Arch Environ Contam Toxicol.

[CR12] Burger J, Tsipoura N, Niles LJ, Gochfeld M, Dey A, Mizrahi D (2015). Mercury, lead, cadmium, arsenic, chromium and selenium in feathers of shorebirds during migrating through Delaware Bay, New Jersey: comparing the 1990s and 2011/2012. Toxics.

[CR13] Carneiro M, Colaço B, Brandão R, Azorín B, Nicolas O, Colaço J, Pires MJ, Agustí S, Casas-Díaz E, Lavín S, Oliveira PA (2015). Assessment of the exposure to heavy metals in griffon vultures (Gyps fulvus) from the Iberian Peninsula. Ecotoxicol Environ Saf.

[CR14] Cheney MA, Hacker CS, Schroder GD (1981). Bioaccumulation of lead and cadmium in the Louisiana heron (*Hydranassa tricolor*) and the cattle egret (*Bubulcus ibis*). Ecotoxicol Environ Saf.

[CR15] Deng H, Zhang Z, Chang C, Wang Y (2007). Trace metal concentration in great tit (*Parus major*) and greenfinch (*Carduelis sinica*) at the Western Mountains of Beijing, China. Environ Pollut.

[CR16] Duker AA, Carranza EJM, Hale M (2005). Arsenic geochemistry and health. Environ Int.

[CR17] Eisler R (1988). Lead hazards to fish, wildlife and invertebrates: a synoptic review, biological report 85.

[CR18] Esselink H, van der Geld FM, Jager LP, Posthuma-Trumpie GA, Zoun PEF, Baars AJ (1995). Biomonitoring heavy metals using the barn owl (*Tyto alba guttata*): source of variation especially relating to body condition. Arch Environ Contam Toxicol.

[CR19] Farahani S, Navid E, Abbasi A, Karimi F, Shiri Malekabad E, Rezaei M (2015). Determination of heavy metals in albumen of hen eggs from the Markazi Province (Iran) using ICP-OES technique. Toxin Rev.

[CR20] Fasola M, Movalli RA, Gandini C (1998). Heavy metals, organochlorine pesticides and PCBs in eggs and feather of heron breeding in northern Italy. Arch Environ Contam Toxicol.

[CR21] Grúz A, Déri J, Szemerédy G, Szabó K, Kormos É, Bartha A, Lehel J, Budai P (2018). Monitoring of heavy metal burden in wild birds at eastern/north-eastern part of Hungary. Environ Sci Pollut Res.

[CR22] Hui CA (2002). Concentrations of chromium, manganese, and lead in air and in avian eggs. Environ Pollut.

[CR23] Jackson A, Evers D, Etterson M, Condon A, Folsom S, Detweiler J, Schmerfeld J, Cristol DA (2011). Mercury exposure affects the reproductive success of a free-living terrestrial songbird, the Carolina wren (*Thryothorus ludovicianus*). Auk.

[CR24] Jayakumar R, Muralidharan S (2011). Metal contamination in select species of birds in Nilgiris District, Tamil Nadu, India. Bull Environ Contam Toxicol.

[CR25] Kim J, Oh J-M (2014). Concentration of trace elements in feathers of waterfowl, Korea. Environ Monit Assess.

[CR26] Kitowski I, Sujak A, Wiącek D, Strobel W, Komosa A, Stobiński M (2016). Heavy metals in livers of raptors from eastern Poland - the importance of diet composition. Belg J Zool.

[CR27] Laskowski R (1991). Are the top carnivores endangered by heavy-metal biomagnification?. Oikos.

[CR28] Lee DP (2003). Lead and cadmium accumulation levels in Korean raptors. Kor J Orni.

[CR29] Legagneux P, Suffice P, Messier JS, Lelievre F, Tremblay JA, Maisonneuve C, Saint-Louis R, Bêty J (2014). High risk of lead contamination for scavengers in an area with high moose hunting success. PLoS One.

[CR30] Lodenius M, Solonen T (2013). The use of feathers of birds of prey as indicators of metal pollution. Ecotoxicology.

[CR31] Mandal P (2017). An insight of environmental contamination of arsenic on animal health. Emerg Contamin.

[CR32] Markowski M, Kaliński A, Skwarska J, Wawrzyniak J, Bańbura M, Markowski J, Zieliński P, Bańbura J (2013). Avian feathers as bioindicators of exposure to heavy metal contamination food. Bull Environ Contam Toxicol.

[CR33] Mashroofeh A, Bakhtiari AR, Ghobeishavi A, Ahmadpour M, Asadi A, Ahmadpour M, Hosseini SH, Eskandari T, Burger J (2015). Mercury levels in avian feathers from different trophic levels of eight families collected from the northern region of Iran. Environ Monit Assess.

[CR34] Mateo R, Guitart R (2003). Heavy metals in livers of water birds from Spain. Arch Environ Contam Toxicol.

[CR35] Monteiro LR, Furness RW (1995). Seabirds as monitors of mercury in the marine environment. Water Air Soil Pollut.

[CR36] Monteiro LR, Granadeiro JP, Furness RW (1998). Relationship between mercury levels and diet in Azores seabirds. Mar Ecol Prog Ser.

[CR37] Movalli PA (2000). Heavy metal and other residues in feathers of laggar falcon Falco biarmicus jugger from six district of Pakistan. Environ Pollut.

[CR38] Naccari C, Cristani M, Cimino F, Arcoraci T, Trombetta D (2009). Common buzzards (Buteo buteo) bio-indicators of heavy metals pollution in Sicily (Italy). Environ Int.

[CR39] Nemery B (1990). Metal toxicity and the respiratory tract. Eur Respir J.

[CR40] Nighat S, Aqbal S, Nadeem MS, Mahmod T, Shah SI (2013). Estimation of heavy metal residues from the feathers of Falconidae, Accipitridae, and Strigidae in Punjab, Pakistan. Turk J Zoo.

[CR41] Nordberg GF (1971). Effects of acute and chronic cadmium exposure on the testicles of mice. Environ Physiol Biochem.

[CR42] NRC (National Research Council) (2005). Mineral tolerance of animals.

[CR43] Pain DJ, Sears J, Newton T (1995). Lead concentrations in birdsof prey in Britain. Environ Pollut.

[CR44] Roque I, Lourenço R, Marques A, Coelho JP, Coelho C, Pereira E, Rabaça JE, Roulin A (2016). Barn owl feathers as biomonitors of mercury: sources of variation in sampling procedures. Ecotoxicology.

[CR45] Rubio I, Martinez-Madrid M, Méndez-Fernández L, Galarza A, Rodriguez P (2016) Heavy metal concentration in feathers of little egret (Egretta garzetta) nestlings in three coastal breeding colonies in Spain. Ecotoxicology 25:30–40 10.1007/s10646-015-1563-0 26467806

[CR46] Scheuhammer AM (1987). The chronic toxicity of aluminium, cadmium, mercury, and lead in birds: a review. Environ Pollut.

[CR47] Scheuhammer AM, Meyer MW, Sandheinrich MB, Murray MW (2007). Effects of environmental methylmercury on the health of wild birds, mammals, and fish. Ambio.

[CR48] Solonen T, Lodenius M (1990). Feathers of birds of prey as indicators of mercury contamination in southern Finland. Holarct Ecol.

[CR49] Spahn SA, Sherry T (1999). Cadmium and Lead exposure associated with reduced growth rates, poorer fledging success of little blue heron chicks (*Egretta caerulea*) in South Louisiana wetlands. Environ Contam Toxicol.

[CR50] Stoica A, Katzenellenbogen BS, Martin MB (2000). Activation of estrogen receptor α by the heavy metal cadmium. Mol Endocrinol.

[CR51] Van Assche FJ (1998) A stepwise model to quantify the relative contribution of different environmental sources to human cadmium exposure. NiCad '98, Prague, Czech Republic, September 21-22, 1998

[CR52] Walsh PM, Furness RW, Rainbow PS (1990). The use of seabirds as monitors of heavy metals in the marine environment. Heavy metals in the marine environment.

[CR53] Wayland M, Bollinger T (1999). Lead exposure and poisoning in bald eagles and golden eagles in the Canadian prairie provinces. Environ Pollut.

[CR54] Webb JL (1966). Enzymes and metabolic inhibitors.

[CR55] Wiemeyer S, Lamont T, Locke L (1980). Residues of environmental pollutants and necropsy data for eastern United States ospreys, 1964-1973. Estuaries.

[CR56] Yamac E, Ozden M, Kirazli C, Malkoc S (2019). Heavy-metal concentrations in feathers of cinereous vulture (*Aegypius monachus L.*) as an endangered species in Turkey. Environ Sci Pollut Res.

[CR57] Zaccaroni A, Amorena M, Naso B, Castellani G, Lucisano A, Stracciari GL (2003). Cadmium, chromium and lead contamination of *Athene noctua*, the little owl, of Bologna and Parma, Italy. Chemosphere.

[CR58] Zarrintab M, Mirzaei R (2018). Tissue distribution and oral exposure risk assessment of heavy metals in an urban bird: magpie from Central Iran. Environ Sci Pollut Res Int.

[CR59] Zarrintab M, Mirzaei R, Mostafaei G, Dehghani R, Akbari H (2016). Concentrations of metals in feathers of magpie (Pica Pica) from Aran-O-Bidgol city in Central Iran. Bull Environ Contam Toxicol.

